# Supplementary figures and images for: Respiratory and intestinal epithelial cells exhibit differential susceptibility and innate immune responses to contemporary EV-D68 isolates
Source: eLife. 2021 Jul 1;10:e66687. doi: 10.7554/eLife.66687 (PMC8285104; doi:10.7554/eLife.66687)

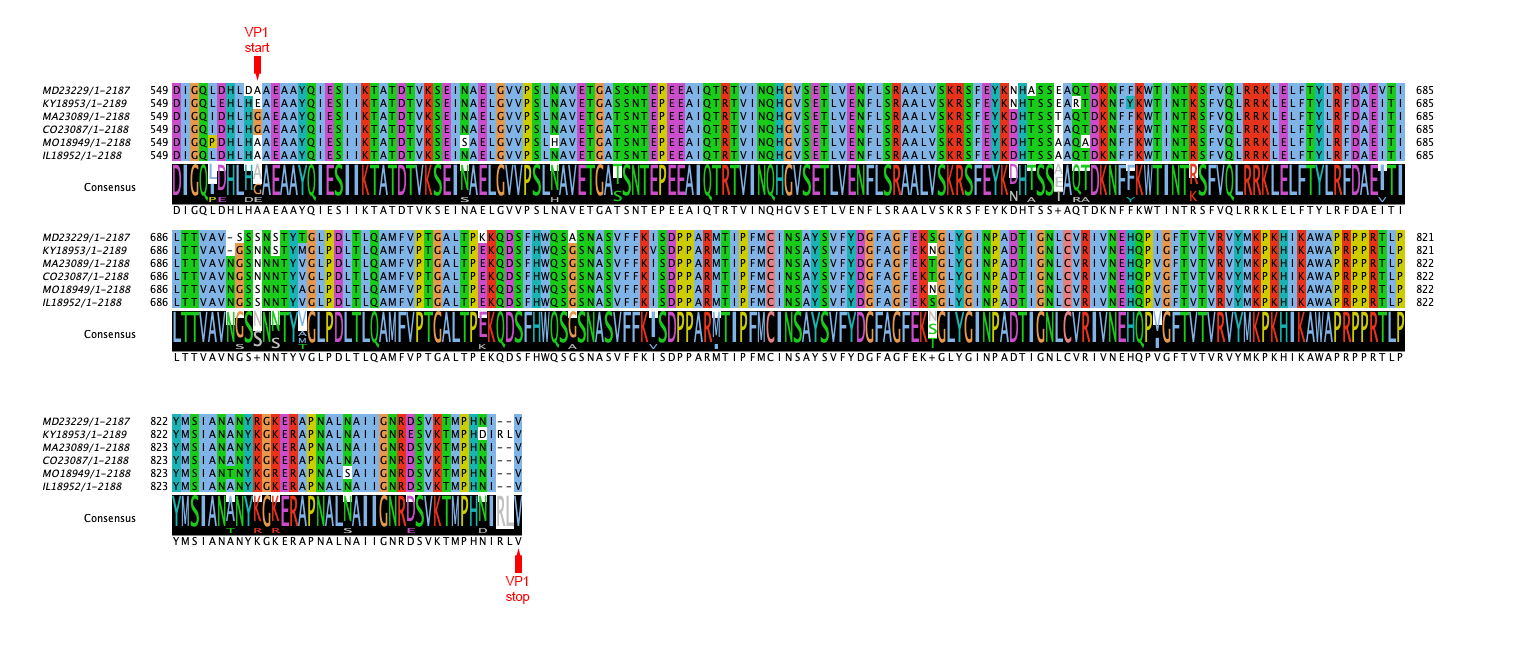

Supplement: Supplementary file 1. — Protein sequence alignment of the VP1 isolates used in the study, with MD/09–23229 used as the reference isolate (shown in top row). The consensus amino acid is denoted in the bottom row. Alignment constructed using Jalview with sequence information from GenBank. [file elife-66687-supp1.zip › Supplementaryfile1.ComparisonofVP1sequences.tif.tif]
