# Supplementary material for: Respiratory and intestinal epithelial cells exhibit differential susceptibility and innate immune responses to contemporary EV-D68 isolates
Source: eLife. 2021 Jul 1;10:e66687. doi: 10.7554/eLife.66687 (PMC8285104; doi:10.7554/eLife.66687)
Supplement: Supplementary file 2. [file elife-66687-supp2.docx]

**Supplemental Table 1**: List of viral isolates used in the study

| Species | Isolate name | Abbreviation | D68 clade | Accession # | Source |
| --- | --- | --- | --- | --- | --- |
| Enterovirus D68 | USA/MD/2009-23229 | MD/09/23229 | A1 | MN240505 | CDC |
| Enterovirus D68 | US/MO/14-18949 | MO/14/18949 | B1 | [KM851227](https://www.ncbi.nlm.nih.gov/nuccore/KM851227) | CDC |
| Enterovirus D68 | US/IL/14-18952 | IL/14/18952 | B2 | [KM851230](https://www.ncbi.nlm.nih.gov/nuccore/KM851230) | CDC |
| Enterovirus D68 | USA/CO/2018-23087 | CO/18/23087 | B3 | [MN245981](https://www.ncbi.nlm.nih.gov/nuccore/MN245981) | CDC |
| Enterovirus D68 | USA/MA/2018-23089 | MA/18/23089 | B3 | [MN245983](https://www.ncbi.nlm.nih.gov/nuccore/MN245983) | CDC |
| Enterovirus D68 | US/KY/14-18953 | KY/14/18953 | D1 | [KM851231](https://www.ncbi.nlm.nih.gov/nuccore/KM851231) | ATCC (VR-1825) |
| Enterovirus 71 | 1095 | EV-A71 | N/A | U22521.1 | ATCC  (VR-784) |
| Echovirus 11 | Gregory | E11 | N/A |  | ATCC  (VR-41) |
