# Supplementary material for: Respiratory and intestinal epithelial cells exhibit differential susceptibility and innate immune responses to contemporary EV-D68 isolates
Source: eLife. 2021 Jul 1;10:e66687. doi: 10.7554/eLife.66687 (PMC8285104; doi:10.7554/eLife.66687)
Supplement: Supplementary file 3. [file elife-66687-supp3.docx]

**Supplemental Table 2**: RT-qPCR Primers

| Primer target | Sequence-F 5’-3’ | Sequence-R 3’-5’ |
| --- | --- | --- |
| CXCL10 | AAAGCAGTTAGCAAGGAAAG | TCATTGGTCACCTTTTAGTG |
| IFIT1 | CAACCAAGCAAATGTGAGGA | AGGGGAAGCAAAGAAAATGG |
| actin | ACTGGGACGACATGGAGAAAAA | GCCACACGCAGCTC |
